# The Impact of Planting Industry Structural Changes on Carbon Emissions in the Three Northeast Provinces of China

**DOI:** 10.3390/ijerph18020705

**Published:** 2021-01-15

**Authors:** Hongpeng Guo, Sidong Xie, Chulin Pan

**Affiliations:** College of Biological and Agricultural Engineering, Jilin University, 5988 Renmin Street, Changchun 130022, China; ghp@jlu.edu.cn (H.G.); xiesd19@mails.jlu.edu.cn (S.X.)

**Keywords:** three provinces in Northeast China, low-carbon agriculture, plantation structure, carbon emissions, net carbon sinks

## Abstract

This paper focuses on the impact of changes in planting industry structure on carbon emissions. Based on the statistical data of the planting industry in three provinces in Northeast China from 1999 to 2018, the study calculated the carbon emissions, carbon absorptions and net carbon sinks of the planting industry by using crop parameter estimation and carbon emissions inventory estimation methods. In addition, the multiple linear regression model and panel data model were used to analyze and test the carbon emissions and net carbon sinks of the planting industry. The results show that: (1). The increase of the planting area of rice, corn, and peanuts in the three northeastern provinces of China will promote carbon emissions, while the increase of the planting area of wheat, sorghum, soybeans, and vegetables will reduce carbon emissions; (2). Fertilizer application, technological progress, and planting structure factors have a significant positive effect on net carbon sinks, among which the changes in the planting industry structure have the greatest impact on net carbon sinks. Based on the comprehensive analysis, it is suggested that, under the guidance of the government, resource endowment and location advantages should be given full play to, and the internal planting structure of crops should be reasonably adjusted so as to promote the development of low-carbon agriculture and accelerate the development process of agricultural modernization.

## 1. Introduction

As one of the significant agricultural development models in achieving the reduction of carbon emissions, low-carbon agriculture has been receiving extensive attention from scholars at home and abroad. This was especially the case in the early days of China’s reform and opening up, with enormous energy consumption and environmental costs accompanying the promotion of rapid economic growth [1]. However, along with the progress of society and the improvement in people’s living standards, the low-carbon agricultural development concept has been recognized by a growing number of people. Nowadays, countries worldwide are encouraging low-carbon development [2], which is aimed at correctly handling the balance between economic growth and the ecological environment on the one hand, while mitigating the adverse effects of global warming on the other. The Intergovernmental Panel on Climate Change (IPCC) indicated in its Fourth Assessment Report that agriculture is currently the second largest source of greenhouse gases, and China’s agriculture accounts for about 11% of the country’s total greenhouse gas emissions [3]. Although agriculture has been a significant source of greenhouse gas emissions, there is a huge potential for its reduction. Therefore, the vigorous development of low-carbon agriculture can be one of the important means to achieve greenhouse gas emission reduction in China [4].

The three provinces of Northeast China are Jilin Province, Heilongjiang Province and Liaoning Province. These three provinces are the main production areas of China’s grain crops [5], among which rice, wheat, corn, soybeans, sorghum, peanuts, and vegetables are the main crops. According to the conditions of the three provinces, Jilin Province has the greatest potential for increasing the production of grain crops, and its net increase in production ranks first in China. According to the 2019 report, grain production increased by 2.45 million tons [6]. Theper capita arable land area and per capita crop yield of Heilongjiang Province ranks first in China [7], and Liaoning Province is an important base for high-quality spring corn [8]. On the whole, the total agricultural area of the three northeastern provinces of China is close to 25 million hectares, accounting for about 15% of China’s total agricultural area [5], and the total grain production accounts for about 20% of China’s total agricultural production. The carbon emissions generated in the process of agricultural production account and about 10.7% of China’s total agricultural carbon emissions [9]. Moreover, the three northeastern provinces have one of the world’s three golden corn belts and one of the only four black soil areas in the world, which shows that the development of crop production here plays a vital role in the development of China’s agriculture. However, the three northeast provinces share the problem of similar irrational planting structures, which has a serious restrictive effect on agricultural carbon emission reduction. Therefore, this article selects the three northeastern provinces as the main research areas and analyzes the impact of planting industry structure changes on carbon emissions by analyzing crop planting area and yield changes and how to adjust the planting industry structure as a new way to promote the development of low-carbon agriculture. At present, under the guidance of the national macro policy, the planting industry structure is evolving in a reasonable direction, which is of great significance to the integration of agricultural resources in the three northeast provinces, the adjustment of the planting industry structure, and the acceleration of low-carbon agricultural development. In addition, this article summarizes domestic and foreign research experience and proposes strategies that are in line with the development of low-carbon agriculture in the three provinces of Northeast China to promote its development.

## 2. Literature Review 

### 2.1. Sources of Agricultural Carbon Emissions

Agriculture is the second largest source of greenhouse gases. Scholars mainly study the sources of carbon emissions from three aspects. The first island use, and deforestation in the process of land use to increase the planting area of agricultural land, which leads to changes in greenhouse gases and climate. The second aspect is the agricultural production process, as due to the input of production factors, including some fossil fuels such as diesel, and the consumption of natural material resources, a large amount of carbon dioxide is emitted. The third aspect is that the burning of the remaining straw after agricultural production leads to a large amount of carbon dioxide emissions.

Some scholars believe that agricultural land use is the main source of agricultural greenhouse gases. According to the relevant report of the IPCC in 2019, the carbon emissions generated in the process of agricultural land use account for about 23% of the total agricultural carbon emissions [10], which is also one of the important reasons leading to global warming and extreme weather. Moreover, the carbon dioxide generated in the process of agricultural land use accounts for 20% of the total agricultural greenhouse gas [11]. Agriculture, forestry and other land use sectors generate about 10% of the global anthropogenic greenhouse gas emissions [12], so agricultural land use is an important factor leading to changes in agricultural carbon emissions [13].

In addition, some scholars believe that the input of production factors in the agricultural production process is also a main contributor to carbon emissions. For example, increasing the application of nitrogen fertilizer can indirectly increase greenhouse gas emissions. The use of chemical fertilizers [14], agricultural irrigation [15], agricultural machinery [16], and agricultural diesel [17] are all major causes of carbon emissions in the process of crop production. Therefore, agricultural production activities are also an important source of greenhouse gases [18]. Many studies have shown that greenhouse gas emissions mainly caused by agricultural production should be reduced [19].

Some scholars believe that after the harvest of crops, the open burning of the remaining straw will also produce a significant amount of greenhouse gas emissions and contribute to global warming [20], which is also an important reason for the deterioration of China’s agricultural environment [21]. In particular, the process of open burning of straw is difficult to control in China. A large amount of carbon dioxide is released into the atmosphere in a short time, and the harmful gases produced at the same time will seriously deteriorate air quality and affect human health [22,23]. Therefore, straw burning is also an important cause of agricultural carbon emissions [24]. Only through reasonable control of straw burning or increased use of straw can the carbon emissions generated by straw burning be reduced.

### 2.2. Measurement of Agricultural Carbon Emissions

At present, both local and international scholars have applied, directly or indirectly, various calculation methods to measure carbon dioxide emissions from the cultivation of various crops, and energy consumption in the agricultural production process. On the basis of the five aspects of agricultural inputs, rice cultivation, livestock and poultry farming, soil, and straw burning, some scholars have measured carbon emissions from agriculture as a whole to quantify the carbon emissions released from various processes in agricultural production [25,26]. Another group of scholars has estimated the agricultural carbon emissions associated with crop type and farming practices by using the plantation crop parameter estimation method for the main carbon emission sources such as fertilizers and pesticides, which are factors of agricultural inputs [27,28].

On top of that, there are more novel methods for measuring agricultural carbon emissions. For example, in order to calculate the total carbon dioxide emissions from the agricultural sector in each country, Dace and Blumberga (2016) created derived indicators and applied multi-criteria analysis methods which are designed to measure the overall magnitude of the climate impact of agricultural carbon emissions [29]. Peter et al. (2017) utilized an environmental assessment calculator for the measurement of greenhouse gas emissions from agricultural products [30]. Mittenzwei (2020) calculated greenhouse gas emissions systematically for different types and sizes of farms in 32 regions with the use of the Jordmod model [31]. Using farmer survey data and factor coefficients, Chen et al. (2020) estimated the carbon footprint of farmers’ agricultural production with a multisystem boundary scenario approach [32]. With a view towards more precise measurement of total carbon emissions from agriculture, Abbas et al. (2020) argued that it is necessary to take back into account post-land use change agricultural practices and soil erosion when calculating carbon footprints [33].

Although there are many different ways to measure carbon emissions from agriculture, the crop parameter estimation method has been widely applied and provides a more accurate way to calculate the emissions from plantation production. Hence, this paper employs the plantation crop parameter estimation method.

### 2.3. Factors Influencing Agricultural Carbon Emissions

Given the increasing awareness of carbon emissions, it is essential to analyze and understand the influencing factors behind carbon dioxide emissions for their reduction. The most direct influencing factor is the input from agricultural production. For examples, agricultural irrigation practices could greatly influence greenhouse gas emissions, and greenhouse gas emissions could be lowered by reduced or water-efficient irrigation [34]. In turn, it is possible that the use of agricultural machinery can significantly increase the carbon emissions of a region [35]. Carbon emissions will also be affected by the inputs of fertilizers, pesticides, agricultural films, agricultural diesel and other elements in the agricultural production process.

Apart from the factor of production inputs, there are also many indicators reflecting a relationship with carbon emissions at the macro level. Among them, an increase in Gross Domestic Product (GDP) and financial expansion will result in an increase in carbon emissions [36,37], while an increase in agricultural output will lead to a decrease in CO_2_ emissions [38]. Therefore, economic factors are one of the main reasons for the increase of greenhouse gas emissions, whose influence trend is mainly an inverse U-shaped nonlinear effect of “promote first and constrain subsequently” [39]. There are also some scholars who have added the influence of energy factors [40,41], productivity factors [42,43], labor factors, agricultural structure, and the carbon intensity of agriculture and livestock [44] to the analysis of economic factors through the LMDI model (Logarithmic Mean Divisia Index) or Kaya identity, which differ in the degree of influence they place on carbon emissions from agriculture. However, beyond the above influencing factors, especially in China, population and income are also important causes of carbon emission changes [45,46].

The role of various influencing factors on carbon emissions has been studied by many domestic and international scholars through LMDI, Kaya and other models to analyze energy factors, economic development levels, production factor inputs, production efficiency and other factors. In particular, the increase in energy consumption, economic growth, and per capita income level show a positive influence on the generation of carbon emissions. This is mainly due to the fact that the economic development of most countries requires the consumption of large amounts of material energy causing a direct increase in carbon emissions. The increase in agricultural output, production efficiency factors, etc., has a suppressing effect on carbon emissions, since advanced technology and rational policies will increase agricultural efficiency and thus indirectly reduce carbon emissions.

### 2.4. Agricultural Carbon Reduction Approaches

With the growing issue of carbon emissions, it has become a focus of attention of scholars at home and abroad as to how to better promote carbon emission reduction. From the perspectives of different agricultural development models, scholars have proposed various measures to reduce carbon emissions. On the one hand, from the aspects of land use and farming patterns, the Irish government has committed to offset 5.6% of its carbon emissions from the increased carbon sink in biomass, soils and land use change for the purpose of reducing carbon emissions [47]. Evans et al. (2015) recommended that carbon emissions can be offset through reforestation methods which can help protect Australia’s biodiversity and mitigate climate change [48]. Dumortier et al. (2020) suggested that the use of biochar in combination with biofuels could indirectly reduce greenhouse gas emissions caused by land use change in the United States [49]. Meanwhile, it has been discovered by other scholars that carbon dioxide emissions from soil can be reduced by optimizing land management, by changing land use patterns [50], or by changing agricultural production and agroforestry intercropping mode [51,52]. It is also possible to significantly reduce agricultural greenhouse gas emissions through the use of climate-smart or conservation agriculture models with existing technologies [53,54].

When it comes to crop cultivation, rice cultivation is also an important source of carbon emissions. Agricultural carbon emissions can be reduced by practicing water-efficient rice cultivation, increasing the use of renewable energy, and adjusting the ratio and area of rice cultivation [55,56]. For the time being, in the face of the current situation of agricultural carbon emissions, China should continue to increase subsidies to agriculture on the basis of learning from the carbon reduction measures of developed countries. At the same time, it can make comprehensive use of various resources at multiple levels to improve the efficiency of resource utilization, so as to further reduce the carbon emissions generated in the agricultural production process and rapidly promote the development of low-carbon agriculture in China.

In a nutshell, the sources of agricultural carbon emissions are mainly generated from the use of soil and from the agricultural production process. The different sources of carbon emissions also result in different methods of calculating carbon emissions and influencing factors. However, there is still a lack of effective and operable countermeasures for agricultural carbon reduction, while the actual situation varies between countries and regions, and therefore the proposed countermeasures and solutions should also be adapted to local conditions. This paper builds on previous research and gives more attention to the impact of changes in plantation structure on carbon emissions. Moreover, at present, in the context of China’s large population, relatively insufficient per capita resources and slow agricultural modernization, the plantation industry is an important constituent of Chinese agriculture. Necessary adjustments to the structure of the plantation industry should be made without reducing China’s grain supply, which is an indispensable key element and the focus of this study. In addition, available studies are mostly focused on a particular province or nationwide, with few studies at the regional level. Furthermore, as a major grain crop export region in China, it is of great significance to study the changes in the structure of the plantation industry in the three northeastern provinces to examine the impact of carbon emissions from the plantation industry.

## 3. Materials and Methods 

### 3.1. Data Sources

The planting industry in this article mainly refers to the seven crops of rice, wheat, corn, soybean, sorghum, peanut, and vegetables in the three northeast provinces. Since the planting area of these seven crops accounts for about 90% of the total agricultural area of these provinces, the output of the seven crops accounts for more than 95% of the total crop production, so this article uses these seven crops to represent the cropping industry.

The time frame of the selected data for this study is 1999–2018. The data on the planting area and the output of the seven major crops in the three northeastern provinces of China are compiled from the China Statistics Bureau, the “Statistical Yearbook” and the “Rural Statistical Yearbook” of each province. The data on the total carbon absorption of crops is calculated based on the crop parameter estimation method of the planting industry. The total carbon emission calculation includes the carbon emissions generated during the agricultural production process and the carbon emissions generated by the burning of crop stalks.

### 3.2. Data Description

According to Table 1, it can be seen that the corn planting area is the largest among the seven crops. The largest corn planting area was 14.5346 million hectares in 2015, and the smallest was 5.4211 million hectares in 2000. Rice and soybeans are also main grain crops in the three northeast provinces. Therefore, the planting area of rice and soybeans is lower than that of corn, but higher than that of wheat, sorghum, peanuts, and vegetables. Among them, the largest sown area of rice was 5.2624 million hectares in 2017, and the smallest was 2.3325 million hectares in 2003; the largest sown area of soybeans was 4.7689 million hectares in 2008, and the smallest was 2.6668 million hectares in 1999. The planting area of wheat dropped from 1.1739 million hectares in 1999 to 0.1130 million hectares in 2018. This is because the subsidies for planting wheat are less, and the income from wheat is lower. Farmers choose other crops with higher income. Compared with 1999, the planting area of sorghum and vegetables in 2018 has also decreased significantly. This is mainly due to higher government subsidies for planting soybeans and corn. Many farmers choose to plant soybeans and corn to increase economic benefits. The main purpose of peanuts is for their oil and high nutritional value. This has led to an increase in the planting area of peanuts in recent years, from 0.1297 million hectares in 1999 to 0.5484 million hectares in 2018.

As can be seen in Table 2, the output of corn in 2018 was as high as 84.4483 million tons, followed by rice, vegetables, soybeans, peanuts, and sorghum. The smallest output was 0.3759 million tons of wheat. In the past 20 years, corn production has witnessed the greatest increase. Compared with 1999, corn production in 2018 increased by 45.3840 million tons, whereas vegetable production witnessed the greatest decrease. In 2018, vegetable production decreased by 7.3578 million tons compared with 1999. Wheat production witnessed the greatest decrease. Wheat production in 1999 was 9.56 times that of 2018. Peanut production witnessed the greatest increase. Peanut production in 2018 was 5.52 times that of 1999, followed by a large increase in rice. The rice output is 2.12 times that of 1999. This is due to the increase in market demand for peanuts and rice in the past 20 years. Therefore, farmers have expanded the production of peanuts and rice in order to further increase their economic income to meet market demand. Compared with the five crops of rice, wheat, corn, peanuts and vegetables, the increase or decrease of soybean and sorghum production is not obvious, indicating that the market demand for sorghum and soybeans is relatively stable, which makes farmers’ soybean and sorghum production more stable. According to the data in Table 2, there are two main reasons for the changes in the yield of these seven crops. On the one hand, the improvement of agricultural technology has increased the yield per unit area, which will increase the total output of some crops. On the other hand, combined with Table 1 and Table 2, it can be found that the change in crop planting area is also an important factor leading to the change of crop yield.

### 3.3. Calculation Model

#### 3.3.1. Carbon Absorption Calculation

According to the yields of the seven major crops of rice, wheat, corn, sorghum, soybean, peanut, and vegetables produced in the three northeastern provinces in the past 20 years, as well as the carbon content rate, fruit moisture coefficient, and economic coefficient of different crops, the carbon absorption of the planting industry can be estimated for the past 20 years. The model of carbon absorption estimation is Formula (1):

(1) $$C_{Z}=\sum_{i}\frac{Y_{i}\times C_{i}\times (1-F_{i})}{M_{i}}$$

In Formula (1): $C_{Z}$ is the total carbon absorption of the planting industry, which refers to the total amount of carbon dioxide absorbed by photosynthesis in the production process of crops; $Y_{i}$ is the harvest yield of i crop; $C_{i}$ is the carbon absorption rate of i crop; $F_{i}$ is the fruit moisture coefficient of i crop; $M_{i}$ represents the economic coefficient of i crop, which is the ratio of economic output to biological yield [57]. Table 3 shows the relative coefficient carbon absorption data of crops [58].

#### 3.3.2. Carbon Emission Measurement

Since it is difficult to measure carbon emissions directly, scholars at home and abroad use a variety of calculation methods to indirectly measure the carbon dioxide, produced by the cultivation of various crops, and energy consumption in the agricultural production process. This is generally measured by the input of carbon sources and data on used materials. The degree of carbon emissions can be also measured by calculating the input and output according to the Life Cycle Approach (LCA). In this paper, using the inventory estimation method of carbon emissions from the planting industry, the carbon emission generated in the production process is calculated by using the amount of agricultural resources, such as chemical fertilizer, pesticide, and agricultural machinery, etc. Equation (2) is the calculation model for the carbon emissions produced from the planting industry in the three northeastern provinces:

(2) $$C_{p}=C_{fert}+C_{film}+C_{pest}+C_{dies}+C_{mach}+C_{irri}=\sum_{i=1}^{n}L_{i}\times Q_{i}$$

In Equation (2), $C_{p}$ is the carbon emission during planting industry production; $C_{fert}$ is the carbon emissions of input fertilizers; $C_{film}$ is the carbon emissions of the input agricultural film; $C_{pest}$ is the carbon emissions of input pesticides; $C_{dies}$ is the carbon emissions of agricultural diesel; $C_{mach}$ is the carbon emission generated by the consumption of fossil fuels in the use of the total power of agricultural machinery and the carbon emission generated by the sown area [17]; $C_{irri}$ is the carbon emissions of electricity consumption during irrigation; $L_{i}$ is the input amount of various agricultural materials; and is the carbon conversion coefficient of various agricultural materials. Among them, the carbon emission from total mechanical power is as follows:

$$C_{mach}=(S\times H)+(M\times R)$$

where S is the sown area of crops, H is the carbon emission coefficient of crop planting area, M is the total power of agricultural machinery, and R is the carbon emissions coefficient of the total power of agricultural machinery. The carbon emission coefficient of agricultural production activities is shown in Table 4.

The carbon emission calculation model of open-air straw combustion in Northeast China is as follows:

(3) $$M=\sum_{i=1}^{n}A_{i}\times P_{i}\times F_{i}$$

(4) $$C_{straw}=\sum M\times EF$$

In Formula (3), M is the burning quantity of crop straws; $A_{i}$ is the yield of different kinds of crops; $P_{i}$ is the straw-grain proportion of different kinds of crops, which is the proportion of the crop straw yields and crop economic yields; $F_{i}$ represents the proportion of open-air burning of crop straw, at 35% in Jilin Province and Heilongjiang Province, and 25% in Liaoning Province [64]. In Formula (4), $C_{straw}$ is the total carbon emission of crop straw combustion; and is the carbon dioxide emission factor of straw combustion, 1.515kg/kg [65]. The crop yield/straw ratio ate shown in Table 5 [66].

#### 3.3.3. Measurement of Net Carbon Sink

The carbon sink of the planting industry refers mainly to the ability of crops to absorb and store carbon dioxide from the air. Net carbon sink refers to the total amount of carbon absorbed by crops through photosynthesis, then subtracting the difference between the total carbon emissions from crop straw combustion and crop production. The calculation model of net carbon sink in Northeast China is as follows:

(5) $$C_{N}=C_{Z}-C_{O}$$

In Formula (5), $C_{N}$ is net carbon sink, which is the difference between the total carbon absorption and total carbon emissions; $C_{Z}$ is the total carbon absorption of crops; and is the total carbon emissions during the planting production process and burning of crop straws.

### 3.4. Model Specification

#### 3.4.1. Multiple Linear Regression Model

According to the planting area and total carbon emissions of seven major crops, the impact of different crops on carbon emissions is calculated, and the preliminary multiple linear regression model is as follows:

(6) $$C_{O}=C+\beta_{1}X_{r}+\beta_{2}X_{w}+\beta_{3}X_{c}+\beta_{4}X_{g}+\beta_{5}X_{s}+\beta_{6}X_{p}+\beta_{7}X_{v}+\varepsilon$$

In Formula (6), C is a constant term; β is the coefficient connection between crop planting area and carbon emissions; and ε is the error term. $C_{O}$ is the total carbon dioxide emissions; $X_{r}$ is the planting area of rice; $X_{w}$ is the planting area of wheat; $X_{c}$ is the planting area of corn; $X_{g}$ is the planting area of sorghum; $X_{s}$ is the planting area of soybean; $X_{p}$ is the planting area of peanut; and $X_{v}$ is the planting area of vegetables.

#### 3.4.2. C-D Production Function

In this paper, the Cobb Douglas production function is used to build a panel data model to analyze the influencing factors of net carbon sink. The equation is Formula (7):

(7) $$C_{N}=A_{t}Land_{t}^{\alpha }Fert_{t}^{\lambda }Labor_{t}^{\varphi }Tech_{t}^{\theta }S_{t}^{\gamma }$$

Take the logarithm of both sides to get Equation (8):

(8) $$\ln C_{N}=C_{t}+\alpha lnLand_{t}+\lambda lnFert_{t}+\varphi lnLabor_{t}+\theta lnTech_{t}+\gamma lnS_{t}$$

In Formulas (7) and (8), $C_{N}$ is the net carbon sink, which is the difference between carbon absorption and carbon emission; t represents year; A and C are constants; Land is the input land resources for crop planting areas; Fert is the amount of fertilizer application; Labor is the agricultural labor force; Tech is the number of patent applications granted; and S is the structure of planting industry, which is the proportion of the planting area of grain crops to the total planting area of crops. The significance test was conducted on five variables, including crop planting area, fertilizer application amount, agricultural labor force, patent application amount and planting structure.

## 4. Results

### 4.1. Changes in the Composition of Carbon Absorption and Carbon Emissions from the Planting Industry

#### 4.1.1. Changes in the Composition of Carbon Sequestration in the Plantation Industry

**Figure 1:**
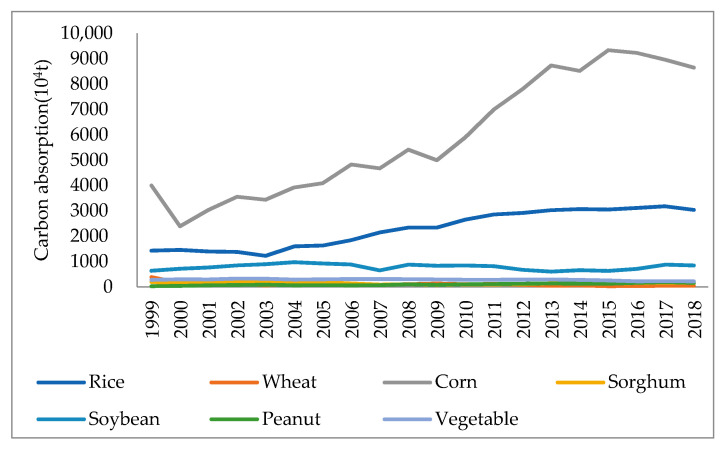
Crop composition of carbon absorption by planting industry. Data Source: calculated by the authors.

As shown in Figure 1, the maximum carbon absorption of corn was 93.3874 million tons in 2015, the maximum carbon absorption of rice was 31.7834 million tons in 2017, and the maximum carbon absorption of soybeans was 9.7030 million tons in 2004. Among them, the main crops that absorb carbon are rice, soybeans, and corn. The main reason is that rice, soybeans and corn are planted in a larger area and their yields will be relatively high. Consequently, rice, soybean and corn also account for the highest percentage of carbon absorption which is the major carbon sink in the plantation industry in the three northeastern provinces. The planted area of the crops has a direct impact on the total carbon absorption. It is necessary to adjust the structure of the plantation industry and re-allocate the plantation ratio of each crop in order to reasonably increase total carbon absorption, so as to speed up the development of low-carbon agriculture in China.

In terms of inter-annual changes, the total carbon absorption of the three crops, including corn, rice and soybean, has a larger proportion, suggesting that the carbon absorption of the plantation industry in the three northeastern provinces mainly relies on these three crops. In particular, the trend of carbon absorption of corn can be roughly categorized into three stages. The first stage is a significant decrease in 1999–2000; the second stage is a fluctuating increase in 2000–2015; followed by a decreasing trend in the third stage between 2016 and 2018. Subsequently, the more significant increase has been in carbon absorption by rice. Carbon absorption by soybean remains more stable and almost unchanged. Since the absolute values of carbon absorption for vegetables, wheat, sorghum and peanuts are relatively small, no significant changes can be seen in the above figure. As compared to corn and grain, the carbon absorption of other crops basically maintains a more stable state.

#### 4.1.2. Changes in the Composition of Carbon Emissions from Straw Burning in the Plantation Industry

**Figure 2:**
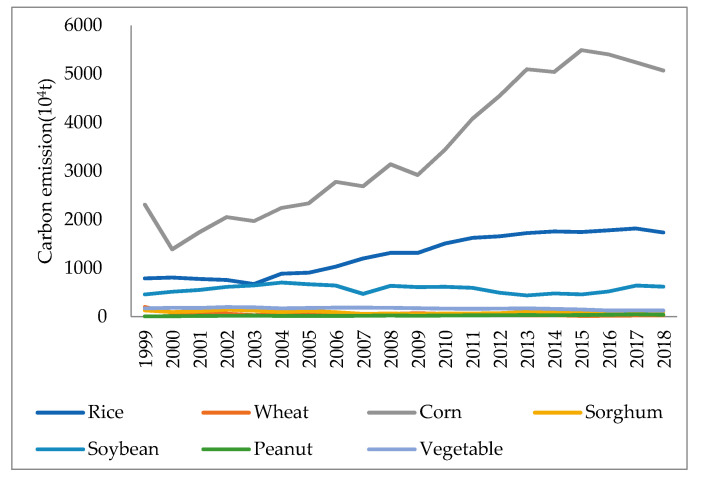
Crop composition of carbon emission from crop straw burning. Data Source: calculated by the authors.

According to Figure 2, the largest carbon emission from the burning of corn stalks was 54.9203 million tons in 2015, the largest carbon emission from the burning of rice straws was 18.1595 million tons in 2017, and the largest carbon emission from the burning of soybean straws were 7.0228 million tons in 2004. Among them, the straw burning of corn, rice, and soybeans is the main source of carbon emissions. On the one hand, this is because the three crops of corn, rice and soybeans grown in the three northeastern provinces have the largest yields, which directly leads to the highest straw production of the three crops. On the other hand, all three crops also have high grass to grain ratios, with low utilization rates of straw, thereby exacerbating the extent of greenhouse gas pollution of the climate and the environment by large amounts of open straw burning. From 1999–2018, the highest percentage of carbon emissions from straw incineration was from corn. The average percentage of carbon emissions from straw burning in the last 20 years has been 59.29%, followed by rice at 22.74%, soybeans at 11.19%, vegetables at 3.35%, sorghum at 1.88%, wheat at 1.16% and peanuts at 0.39%. By food crop, the average percentage of carbon emissions from straw burning amounted to 85.07%, whereas that from cash crop straw burning has amounted to 14.93%.

Although in recent years the three northeastern provinces have increased efforts to control straw burning, and the Ministry of the Environment has also monitored the phenomenon of straw burning in various areas with satellite remote sensing inspections, the prevention and control of pollution from straw burning is still very limited. This is primarily due to the fact that straw crushing and returning back to land is more difficult to implement in the Northeast. The cold winter in the three northeastern provinces makes it challenging for the crushed straw to decompose, thereby creating gaps in the soil where the crushed straw is mixed. The crops that are re-planted will suffer from incomplete contact with the soil and from diseases and insects in the straw, which will result in reduced crop yields and production. There is an urgent need to identify more alternatives for the use of straw in order to reduce the environmental pollution caused by irrational use.

#### 4.1.3. Changes in the Proportion of Carbon Emissions from Inputs to Factor Production in the Plantation Industry

**Figure 3:**
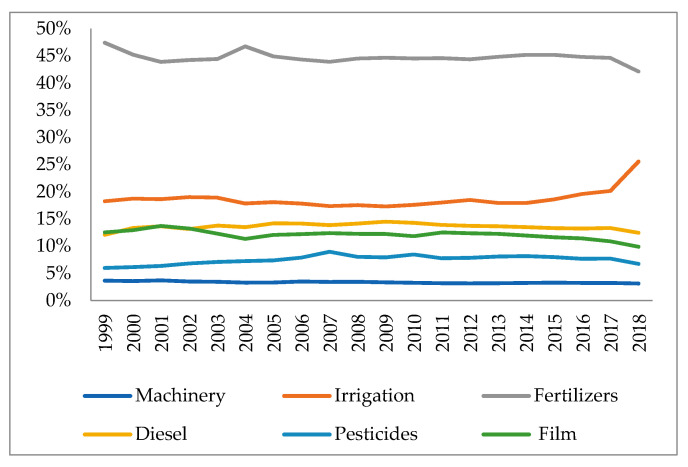
Proportion of carbon emissions from agricultural element inputs in plantation. Data Source: calculated by the authors.

According to Figure 3, it can be observed that the largest proportion of carbon emissions in the plantation production process in the three northeastern provinces is attributable to the use of agricultural fertilizers. Subsequently, the proportion of carbon emissions decreases in order for: the effective irrigated area of plantation, the use of agricultural diesel, agricultural film, pesticides, and total machinery power. Therefore, it is necessary to strengthen the control of the use of chemical fertilizers with reasonable adjustment of usage of the various agricultural materials according to changes in the structure of the plantation industry, so as to reduce the carbon emissions generated. In addition, the increase in the total input of agricultural production factors in the plantation industry is an influential reason for the future growth of carbon emissions. Therefore, it is necessary to rationally manage the use of these agricultural inputs and reduce the carbon emissions generated by these inputs by improving farming techniques or appropriate farming patterns.

As an overall trend, the proportion of carbon emissions from the use of various agricultural materials has remained fairly stable over the recent 2001–2016 period. However, there has been a clear upward trend in carbon emissions from the effective irrigated area under cultivation in the last three years, whereas the rest of the agricultural materials have shown a downward trend. This is likely attributable to the fact that lakes and surface rivers are being over-exploited and continuously polluted by factories and cities in the relatively arid northern regions. Farmers have to exploit groundwater for irrigation, consuming large amounts of energy in the process and generating large amounts of carbon emissions directly and indirectly.

#### 4.1.4. General Characteristics of Carbon Emissions from Plantations

Through the measurement and analysis of carbon sequestration, carbon emissions and net carbon sinks in the three northeastern provinces over the past two decades (1999–2018), the following conclusions can be drawn in this paper.

Firstly, from the overall development trend of the three northeastern provinces, there has been a fluctuating increase in total carbon sequestration, carbon emissions and net carbon sinks. However, the increase in total carbon sequestration is higher than that of carbon emissions. In 2018, the total carbon sequestration increased by 61,463,400 tons and the total carbon emissions increased by 42,296,600 tons compared to 1999, with the growth rate of carbon sequestration being higher than that of carbon emissions each year. As a result, the overall total net carbon sink is on an annual upward trend, increasing from 22,144,300 tons in 1999 to 40,641,100 tons in 2018, which is an 83.53% increase over the past two decades.

Secondly, food crops account for approximately 80–90% of the total carbon sequestration of the plantation industry in the three northeastern provinces, while cash crops account for 10–20%. Among them, food crops such as corn, rice, wheat and sorghum occupy an average of 59.99%, 23.77%, 1.65% and 1.29%, respectively, of the total carbon sequestration over 20 years, while cash crops such as soybeans, peanuts and vegetables occupy an average of 9.06%, 0.96% and 3.29%, respectively.

Lastly, during the past two decades in the three northeastern provinces, the highest carbon emissions are mainly from fertilizer inputs, followed by effective irrigation area, agricultural diesel, agricultural film, pesticides, and total agricultural machinery power. All six of these inputs are the main sources of carbon emissions from the plantation production process. The process of straw burning in the plantation industry is responsible for 70–90% of carbon emissions from food crops and 10–30% from cash crops. Among food crops, the highest carbon emissions have been from the burning of corn straw, which has averaged 59.29% over the past 20 years, followed by rice at 22.74%, sorghum at 1.88% and wheat at 1.16%; among cash crops, soybean straw burning has generated the highest carbon emissions with an average percentage of 11.19%, followed by vegetables at 3.35% and peanuts at 0.39%.

### 4.2. Principal Component Analysis

PCA (Principal Component Analysis) is a commonly used data analysis method. PCA transforms the original data into a set of linearly independent representations of each dimension through linear transformation. It can be used to extract the main feature components of the data and is often used for high-dimensional data reduction, which means PCA can reduce the number of independent variables, thereby eliminating the degree of correlation between independent variables. Since the model has autocorrelation, the first-order difference term $DC_{O}$ of $C_{O}$ is included in the independent variables, and two common factors of the principal components are considered. The model (9) is as follows:

(9) $$C_{O}=1353.5320+0.0934DC_{O}+1.0166F_{1}+0.0568F_{2}$$

Therefore, the adjusted model is (10):

(10) $$\begin{array}{c} C_{O}=1353.5320+0.0934DC_{O}+0.4294Xr-0.3823Xw+0.4249Xc\\ -0.4216Xg-0.0385Xs+0.4175Xp-0.4149Xv \end{array}$$

Since the carbon emissions in this period are affected by carbon emissions in the previous period, the first-order difference term for carbon emissions is introduced with a coefficient of 0.0934. Moreover, the expansion of the planting area of rice, corn, and peanuts will promote carbon emissions; every increased 1000-hectares planting areas will add 4294 tons, 4249 tons, and 4175 tons of carbon emissions, respectively. However, the expansion of wheat, sorghum, soybean, and vegetable prohibit carbon emissions, and every increased 1000-hectares planting area will decrease 3823 tons, 4216 tons, 385 tons, and 4149 tons of carbon emissions.

### 4.3. Empirical Results of Panel Data

#### 4.3.1. Unit Root Test

Before establishing the panel regression model, it is necessary to check the stability of the panel data to avoid the phenomenon of “false regression”. At the same time, the variables are processed logarithmically, in order to reduce data fluctuation and heteroscedasticity. This article uses Augmented Dickey-Fuller (ADF) and Levin-Lin-Chu (LLC) tests, and the results are shown in Table 6 and Table 7.

Among them, lnC_N_ refers to the net carbon sink after taking logarithm, and d.lnC_N_ refers to the first-order difference of net carbon sink after taking logarithm. Similarly, Land is the input land resources for crop planting areas; Fert is the amount of fertilizer application; Labor is the agricultural labor force; Tech is the number of patent applications granted; S is the structure of planting industry, which is the proportion of the planting area of grain crops to the total planting area of crops. The ADF test results are as follows: the ADF values of lnC_N_, lnLand, lnFert, lnTech, lnS, and lnLabor are arranged in order as 5.2214, 1.9251, 4.3543, 0.4810, 3.5068, 1.8976, and the corresponding P values are all bigger than 0.05, therefore all of the variables are unstable. After the first-order difference, the ADF values of lnC_N_, lnLand, lnFert, lnTech, lnS, and lnLabor become 33.1424, 70.1202, 51.9592, 13.8363, 16.2996, and 57.6351, respectively, which all pass the significance test, indicating that the variables are stable and are first-order single integer sequence.

The LLC test results are as follows: The LLC values of lnC_N_, lnLand, lnFert, lnTech, lnS, and lnLabor are arranged in sequence as 1.5015, 0.1046, 3.2568, 1.5218, 0.9111, 1.3505, and the corresponding P values are all bigger than 0.05, therefore all of the variables are unstable. After the first-order difference, the ADF values of lnC_N_, lnLand, lnFert, lnTech, lnS, and lnLabor become −4.7961, 6.0758, −1.9716, −4.1831, −3.3895, respectively, and all passed significance tests, which indicates stable variables, as a first order single integer sequence.

#### 4.3.2. Cointegration Test

According to the unit root test in Table 8, although the data lnC_N_, lnLand, lnFert, lnTech, lnS, and lnLabor are unstable series, they are all first-order single integer I (1), which indicates that there may be a long-term equilibrium relationship between variables. In this paper, the Kao test and Pedroni test are used to test the cointegration of variables. In the Kao test, the ADF value is −1.5740, and the counterpart P value is 0.0577. In the Pedroni test, the Phillips-Perron (PP) statistic and ADF statistic is −1.5420 and −1.4728 respectively, the counterpart *p* value is 0.0615 and 0.0704 respectively, all smaller than 0.1. It shows that there is a long-term balancing connection between variables, which is worthy regression analysis.

#### 4.3.3. Panel Data Regression Results

There are three kinds of panel regression models: mixed regression model, fixed effect model and random effect model. Before regression analysis, the F test and Hausman test are needed to determine the optimal model. First of all, F value is used to judge whether the mixed regression model or fixed effect model should be selected. If we choose the latter, we need to use the Hausman test to determine whether the random effect model or the fixed effect model should be established.

After testing, the value of F statistic is 1.9800, and the corresponding *p* value is 0.1477, which is greater than 0.05, indicating that the mixed regression model should be selected rather than the fixed effect model.

Table 9 is the regression analysis table. R-square is the goodness of fit index, which represents the explanatory power of independent variable to dependent variable. When the value is between 0 and 1, the closer the value is to 1, the higher the independent variable interprets the dependent variable; On the contrary, the closer the value is to 0, the weaker the explanatory power of independent variable to dependent variable.

In Table 9, the value of R-square in the mixed regression model is 0.9106, which means that the independent variable can explain 91.06% of the change in the dependent variable. Firstly, compared with other variables, lnS has the highest regression coefficient, which makes the calculation of net carbon sink more easily affected by the structure of the planting industry. Secondly, chemical fertilizer, labor force, scientific and technological progress, and planting area of crops all have positive effects on net carbon sink. The regression coefficients of lnLand, lnFert, lnTech, lnS, and lnLabor to lnC_N_ are 0.0762, 0.5756, 0.1802, 1.8209, and 0.3943. Among them, it can be seen that lnLand and lnLabor have no significant influence on lnC_N_, while lnTech has significant influence on lnC_N_ at 1% level and lnFert and lnS at 5% level.

## 5. Discussion

According to the results above, several approaches to promote net carbon sinks are as follows. Firstly, changes in the plantation structure have the greatest impact on the net carbon sink, in which the three northeastern provinces are still dominated by grain. Consequently, the plantation structure within grain crops should be rationally regulated to increase the planted area of wheat and sorghum and reduce the planted area of rice and corn while keeping the production of grain crops and the planted area of grain crops intact, so as to reduce total carbon emissions and increase net carbon sinks; in the case of cash crops, priority should be given to increasing the proportion of vegetable crops, since it is more cost-effective to reduce carbon emissions by increasing the area planted with vegetables. Besides, the lower yield of vegetable straw not only alleviates the problem of straw disposal, but also reduces the carbon emissions from straw burning. On the other hand, although soybean also plays an inhibiting role on carbon emissions, the grass-grain ratio of soybean straw is up to 1.6, which makes the benefit of increasing the planted area of soybean to reduce carbon emissions relatively low. There is a contribution of peanuts to carbon emissions reduction; however, the yield of peanuts is low and so is the degree of carbon sequestration. As a result, the economic and ecological benefits of peanuts should be further enhanced by improving peanut yields through advanced planting techniques and rational planting patterns.

Secondly, the increased use of fertilizers in the three northeastern provinces is one of the most important reasons for promoting net carbon sinks. This is because the use of chemical fertilizers can facilitate the growth and development process of crops, where the process can increase the absorption of carbon emissions. However, the negative effects of chemical fertilizers’ usage are still difficult to eliminate, especially the large number of chemical residues after using chemical fertilizers. This not only affects the fertility of the land which has an impact on the re-planting of crops, but also endangers human health, and therefore the use of chemical fertilizers in the plantation production process needs to be strictly controlled.

Thirdly, the advancement of science and technology has significantly contributed to the net carbon sink of the plantation industry in the three northeastern provinces, and this suggests that the development of science and technology is becoming distinctively beneficial to the plantation industry, with increasingly widespread applications. For example, the utilization of advanced planting equipment not only reduces energy consumption but also increases production efficiency; research on new crop varieties greatly enhances the yield and quality of agricultural products; the development of genetically modified crops can reduce pests and diseases, and so on. All of these are favorable changes due to the progress of science and technology, which should continue to increase. It is more important to increase the application of plantation-related technologies, as only when a technology is really put into practice with promising results achieved can the development of low-carbon agriculture and agricultural modernization can be truly accelerated. In the future, if advanced technology or increased use of straw can be used to reduce the amount of straw burning, thereby reducing the carbon emissions from straw burning, it can increase the soil organic carbon sink until a new balance is reached. Therefore, reducing straw burning can also make a positive contribution to the net carbon sink.

Fourthly, it can be seen from the results in Table 9 that the regression coefficients of planted area and labor on net carbon sink are positive, yet the effect is not significant. It is likely to be the problem of sample data limitation that makes the theoretical analysis and empirical phenomenon analysis inconsistent. In terms of inter-annual variation, an increase in planted area does not translate into an increase in total net carbon sinks, and a decrease in planted area does not necessarily translate into a decrease in net carbon sinks. However, considering the overall trend over the last 20 years, both the area planted and the total net carbon sink have generally been increasing. There are two main reasons for the increase of planting area. On the one hand, China has cultivated a lot of wasteland and cut down some forests to increase the planting area of crops. On the other hand, farmers have transformed the planting area of some crops with low economic benefits into the planting area of crops with higher economic benefits, which leads to the increase of planting area of some crops. The labor force reflects the number of people involved in agriculture, where a large number of farmers are now using farm machinery for seeding, substantially increasing productivity. The slow increase in the number of farmers, yet the very rapid increase in crop yields, has also indirectly increased the net carbon sink. Therefore, the impact of changes in planted area and labor on the net carbon sink may not be a simple linear relationship.

## 6. Conclusions

### 6.1. Restructuring of Plantations Industry

From the viewpoint of the crop composition of the plantation industry, it is necessary to moderately reduce the planted area of rice, corn and peanuts, and increase the planted area of wheat, sorghum, soybeans and vegetables to reduce carbon emissions, on the premise of ensuring no reduction in food production. In recent years, the improvement in corn prices and subsidies, as well as the impact of imported soybeans on the China’s soybean market, have driven farmers to plant corn, which is more economically profitable. The significant increase in the planted area of corn and the decrease in the planted area of soybean and wheat crops have contributed to the homogenization of the plantation structure in the three northeastern provinces, which is not conducive to the balanced development of the ecological environment. Despite the increase in the income of farmers in the three northeastern provinces, the ecological environment has not been improved. Therefore, adjustment of the plantation industry structure has yet to be implemented in order to give full play to the advantages of resource endowment and of the geographical location of the three northeastern provinces. It is not advisable to focus only on the immediate benefits, which will hinder the entire development process of low-carbon agriculture and modern agriculture. It is necessary to ensure high-quality development while focusing on reducing the waste of resources, with a view to increasing the overall profitability of low-carbon agriculture. Government guidance and subsidies are also essential for the restructuring of the plantation industry, which can be beneficial to enhance farmers’ motivation. It is also believed that reasonable restructuring of plantations will also produce a greater positive impact on the ecological and social environment.

In conclusion, although there are many factors affecting agricultural carbon emissions, it is easy to neglect the important impact of changes in the structure of the plantation industry. Most scholars tend to consider the impact of agricultural carbon emissions from the perspective of agriculture as a whole, while paying less attention to the changes in its internal plantation structure. Whether it is from the relative change in plantation ratio or the absolute change in plantation carbon emissions, structural change of the plantation industry is one of the significant influencing factors regarding carbon emissions. Consequently, a reasonable plantation structure is not only an effective way to reduce carbon emissions, but also a favorable way to improve the ecological environment.

### 6.2. Limitations and Further Research

Although this study has drawn some meaningful research conclusions, there are still shortcomings. For example, because the research object of this article is only aimed at the carbon dioxide emissions of the planting industry, the production processes in the planting industry can also release other polluting gases such as methane and nitrogen dioxide. Therefore, in future research, it is expected that types of greenhouse gases other than carbon dioxide can be considered, and thus research can be further optimized by collecting more abundant data and covering more influential factors.

## Figures and Tables

**Table 1:** Planting area of different crops in Northeast China.

Year	Rice (10^4^ hm^2^)	Wheat (10^4^ hm^2^)	Corn (10^4^ hm^2^)	Sorghum (10^4^ hm^2^)	Soybean (10^4^ hm^2^)	Peanut (10^4^ hm^2^)	Vegetable (10^4^ hm^2^)
1999	258.16	117.39	670.53	43.99	266.68	12.97	99.51
2000	268.04	78.51	542.11	42.62	370.92	21.56	112.46
2001	276.94	57.59	630.90	37.78	409.20	27.72	109.97
2002	278.69	33.36	629.67	39.22	363.05	30.40	120.41
2003	233.25	27.18	611.59	32.54	412.44	40.48	113.72
2004	273.21	28.70	667.98	23.53	437.73	30.82	95.13
2005	287.27	28.03	678.79	26.96	430.67	30.82	95.67
2006	327.34	25.26	816.89	42.61	461.72	23.29	88.27
2007	360.91	24.99	898.20	26.13	453.06	28.81	80.85
2008	393.18	25.33	880.31	23.61	476.89	38.32	79.20
2009	398.77	30.36	948.36	21.05	476.30	36.18	68.53
2010	445.36	28.83	1024.85	19.72	422.17	40.05	68.19
2011	474.20	30.44	1089.21	17.70	381.79	39.68	73.49
2012	494.13	21.68	1213.93	19.68	319.59	42.24	77.02
2013	517.81	13.91	1298.25	17.56	295.09	43.62	75.44
2014	521.76	15.13	1352.91	19.02	309.92	44.36	73.68
2015	516.64	7.35	1453.46	15.50	290.33	48.39	69.01
2016	520.19	8.19	1356.02	18.78	348.01	56.15	58.98
2017	526.24	10.78	1271.88	20.77	403.00	62.30	59.68
2018	511.12	11.30	1326.23	20.25	392.04	54.84	58.59

Source: China Statistical Yearbook and China Rural Statistical Yearbook.

**Table 2:** Yield of different crops in three provinces of Northeast China.

Year	Rice (10^4^ t)	Wheat (10^4^ t)	Corn (10^4^ t)	Sorghum (10^4^ t)	Soybean (10^4^ t)	Peanut (10^4^ t)	Vegetable (10^4^ t)
1999	1764.84	359.54	3906.43	172.47	549.40	29.41	3660.66
2000	1794.10	147.90	2335.10	124.70	618.50	40.82	3919.01
2001	1722.65	120.83	2966.58	152.29	660.90	60.47	3889.26
2002	1697.20	108.84	3468.50	191.97	736.97	72.89	4309.12
2003	1512.40	51.90	3353.40	161.20	775.70	86.34	4227.52
2004	1969.12	95.27	3829.20	132.87	842.66	65.28	3802.50
2005	2016.00	104.58	3993.40	154.59	798.10	66.51	3940.89
2006	2279.60	99.10	4712.51	117.47	766.86	59.97	4079.35
2007	2652.97	75.26	4562.81	76.75	562.80	70.30	4093.40
2008	2889.46	95.42	5285.17	85.06	759.79	102.10	4047.19
2009	2891.48	120.60	4878.88	63.12	722.32	81.18	3902.69
2010	3279.78	96.22	5760.23	84.20	731.90	106.67	3705.00
2011	3528.99	107.31	6832.09	82.43	706.46	114.29	3681.46
2012	3599.74	72.78	7614.48	89.15	586.53	126.80	3765.08
2013	3735.13	41.57	8527.84	119.90	521.83	139.33	3892.07
2014	3787.90	49.23	8319.12	127.23	568.09	127.88	3639.22
2015	3767.86	23.02	9116.08	110.33	543.71	131.42	3372.14
2016	3844.46	29.85	9009.16	125.27	614.96	167.46	2885.16
2017	3925.81	39.50	8743.33	144.28	758.90	194.26	2953.07
2018	3749.88	37.59	8444.83	132.35	730.90	162.24	2924.88

Source: China Statistical Yearbook and China Rural Statistical Yearbook.

**Table 3:** Carbon absorption coefficient of crops.

Crops	Carbon Absorption Rate	Fruit Moisture Coefficient	Economic Coefficient
Rice	0.414	0.120	0.450
Wheat	0.485	0.120	0.400
Corn	0.471	0.130	0.400
Sorghum	0.450	0.120	0.350
Soybean	0.450	0.130	0.340
Peanut	0.450	0.100	0.430
Vegetable	0.450	0.900	0.600

Source: [58].

**Table 4:** Carbon emission coefficient of agricultural production activities.

Sources	Carbon Emissions Coefficient	References
Irrigation	266.48 kg/hm^2^	[59]
Fertilizers	0.8956 kg/kg	[60]
Diesel	0.5927 kg/kg	[10]
Pesticides	4.9341 kg/kg	[61]
Agricultural films	5.18 kg/kg	[62]
Sown area	16.47 kg/hm^2^	[63]
Agricultural machinery	0.18 kg/kw	[63]

**Table 5:** Ratio of straw to yield of different crops.

Crop	Rice	Wheat	Corn	Sorghum	Soybean	Peanut	Vegetable
Ratio	0.9	1.1	1.2	1.6	1.6	0.5	0.1

Source: [66].

**Table 6:** ADF Unit root test.

Variable	Inspection Form (C,T,K)	ADF Statistics	*p* Value	Stability
lnC_N_	(0,0,1)	5.2214	0.5158	unstable
d.lnC_N_	(0,0,1)	33.1424	0.0000	stable
lnLand	(0,0,1)	1.9251	0.9265	unstable
d.lnLand	(0,0,1)	70.1202	0.0000	stable
lnFert	(0,0,1)	4.3543	0.6289	unstable
d.lnFert	(0,0,0)	51.9592	0.0000	stable
lnTech	(0,0,1)	0.4810	0.9981	unstable
d.lnTech	(0,0,2)	13.8363	0.0315	stable
lnS	(0,0,1)	3.5068	0.7431	unstable
d.lnS	(0,0,2)	16.2996	0.0122	stable
lnLabor	(0,0,1)	1.8976	0.9289	unstable
d.lnLabor	(0,0,0)	57.6351	0.0000	stable

Note: C represents the constant term, T represents the time trend, and K represents the lag order. “ln”in the table refers to the logarithm, “d.” refers to first-order difference.

**Table 7:** LLC Unit root test.

Variable	Inspection Form (C,T,K)	LLC Statistics	*p* Value	Stability
lnC_N_	(C,T,1)	1.5015	0.9334	unstable
d.lnC_N_	(0,0,1)	−4.7961	0.0000	stable
lnLand	(C,T,1)	0.1046	0.5417	unstable
d.lnLand	(C,T,1)	−6.0758	0.0000	stable
lnFert	(0,0,1)	3.2568	0.9994	unstable
d.lnFert	(C,T,1)	−1.9716	0.0243	stable
lnTech	(C,0,1)	1.5218	0.9360	unstable
d.lnTech	(C,T,1)	−4.0168	0.0000	stable
lnS	(C,T,1)	0.9111	0.8189	unstable
d.lnS	(0,0,1)	−4.1831	0.0000	stable
lnLabor	(C,T,1)	1.3505	0.9116	unstable
d.lnLabor	(0,0,1)	−3.3895	0.0004	stable

Note: C represents the constant term, T represents the time trend, and K represents the lag order. “ln”in the table refers to the logarithm, “d.” refers to first-order difference.

**Table 8:** Panel cointegration test.

Test Method	Statistic	Statistical Value	*p* Value
Kao Test	ADF	−1.5740	0.0577
Pedroni Test	Panel PP-Statistic	1.5420	0.0615
	Panel ADF-Statistic	−1.4728	0.0704

**Table 9:** Panel model determination and regression results.

Variable	Model 1	Model 2	Model 3
	OLS	FE	RE
lnLand	0.0762	0.6983 *	0.0762
	[0.5400]	[1.8300]	[0.5400]
lnFert	0.5756 **	0.4454	0.5756 **
	[2.2100]	[1.6200]	[2.2100]
lnTech	0.1802 ***	0.1131 *	0.1802 ***
	[4.0100]	[1.9100]	[4.0100]
lnS	1.8209 **	1.9829 **	1.8209 **
	[2.4600]	[2.6300]	[2.4600]
lnLabor	0.3943	0.2230	0.3943
	[1.2000]	[0.4200]	[1.2000]
Constant	−9.0537 **	−12.8592 **	−9.0537 ***
	[−2.6200]	[−2.6000]	[−2.6200]
Number	60	60	60
R-squared	0.9106	0.8920	0.8840
F	110.0700 ***	85.9300 ***	550.3400 ***
F test: F (2,52) = 1.9800 *p* = 0.1477			

Note: ***, **, and * represent *p* < 0.01, *p* < 0.05 and *p* < 0.1 respectively, and the values in brackets are t values. OLS = ordinary least square model, FE = fixed effect model, RE = random effect model.

## Data Availability

Publicly available datasets were analyzed in this study. This data can be found here: China Statistical Yearbook (http://www.stats.gov.cn/english/Statisticaldata/AnnualData/) and China Rural Statistical Yearbook (https://data.cnki.net/trade/yearbook/single/n2019120190?z=z009).
